# Supplementary material for: A distributed brain response predicting the facial expression of acute nociceptive pain
Source: eLife. 2024 Nov 11;12:RP87962. doi: 10.7554/eLife.87962 (PMC11554303; doi:10.7554/eLife.87962)
Supplement: Supplementary file 1. — (A) Peak regions with positive weights contributing to the prediction of the facial expression scores. (B) Peak regions with negative weights contributing to the prediction of the facial expression scores. (C) Mixed-effect model results for the effect of pain ratings on the logarithmic transformed facial action coding system (FACS) scores. (D) Mixed-effect model results for the effect of runs and trials on the logarithmic transformed FACS scores. (E) Mixed-effect model results for the effect of runs and trials on the pain ratings. (F) Mixed-effect model results for the effect of the facial expression of pain signature (FEPS) expression scores and the pain ratings on the logarithmic transformed FACS scores. [file elife-87962-supp1.docx]

**Supplementary file 1A.** Peak regions with positive weights contributing to the prediction of the facial expression scores.

| **Regions** | **x** | **y** | **z** | **Clusters size**  **(mm^3^)** | **Peaks**  **z-score** |
| --- | --- | --- | --- | --- | --- |
| Frontal pole (bilateral) | -10 | 70 | -2 | 1928 | 4.43 |
| Lateral inferior frontal gyrus (bilateral) | -42 | 60 | -8 | 336 | 4.78 |
| Precentral gyrus (M1; right) | 44 | -14 | 38 | 3640 | 5.96 |
| Precentral gyrus (M1; left) | -46 | -10 | 36 | 2472 | 4.90 |
| Parietal operculum (right) | 48 | -26 | 34 | 256 | 3.58 |
| Inferior parietal lobule (right) | 56 | -28 | 42 | 56 | 3.20 |
| Posterior cingulate gyrus (right) | 20 | -40 | 46 | 304 | 3.90 |
| Occipitotemporal/ Inferior temporal gyrus (left) | -48 | -80 | 8 | 168 | 3.96 |

The main clusters are shown in Figure 1

**Supplementary file 1B.** Peak regions with negative weights contributing to the prediction of the facial expression scores.

| **Regions** | **x** | **y** | **z** | **Clusters size**  **(mm^3^)** | **Peaks**  **z-score** |
| --- | --- | --- | --- | --- | --- |
| vlPFC (right) | 36 | 52 | -2 | 1128 | -4.09 |
| vlPFC (left) | -40 | 50 | -2 | 1288 | -4.71 |
| dlPFC (right) | 42 | 46 | 20 | 1136 | -4.06 |
| dlPFC (left) | -38 | 40 | 14 | 688 | -4.04 |
| vmPFC/subgenual ACC (bilateral) | -4 | 34 | -10 | 5560 | -4.80 |
| aMCC | 0 | 20 | 30 | 24 | -3.13 |
| Ventral striatum | 14 | 18 | -10 | 536 | -3.94 |
| Superior frontal gyrus | 24 | 10 | 58 | 168 | -3.43 |
| Temporal operculum (right) | 52 | 8 | -6 | 152 | -4.00 |
| Middle frontal gyrus (premotor area; left) | -32 | 0 | 58 | 160 | -3.71 |
| MCC (right) | 4 | 4 | 44 | 16 | -3.27 |
| Post central (S1)/inferior parietal lobule (right) | 50 | -36 | 50 | 88 | -3.37 |
| Fusiform gyrus (right) | 28 | -60 | -14 | 56 | -3.45 |
| Precuneus (left) | -14 | -66 | 42 | 544 | -4.08 |
| Precuneus (right) | 12 | -66 | 54 | 48 | -3.31 |

The main clusters are shown in Figure 1

**Supplementary file 1C.** Mixed-effect model results for the effect of pain ratings on the logarithmic transformed FACS scores.

|  | **Log(FACS scores + 1)** | | |
| --- | --- | --- | --- |
| *Predictors* | *Estimates* | *CI* | *p* |
| (Intercept) | 0.99 | 0.66 – 1.33 | <.001 |
| Pain ratings | 0.08 | -0.01 – 0.21 | .069 |
| **Random Effects** |  |  |  |
| σ^2^ | 0.36 |  |  |
| τ_00 Subject_ | 0.95 |  |  |
| τ_11 Subject.Rating_ | 0.05 |  |  |
| *ρ*_01 Subject_ | 0.64 |  |  |
| ICC | 0.74 |  |  |
| N _Subject_ | 34 |  |  |
| Observations | 533 |  |  |
| Marginal R^2^ / Conditional R^2^ | 0.008 / 0.737 |  |  |

**Supplementary file 1D.** Mixed-effect model results for the effect of runs and trials on the logarithmic transformed FACS scores.

|  | **Log(FACS scores + 1)** | | |
| --- | --- | --- | --- |
| *Predictors* | *Estimates* | *CI* | *p* |
| (Intercept) | 1.00 | 0.63 – 1.37 | <.001 |
| Trial | -0.00 | -0.03 – 0.03 | .902 |
| Run | 0.02 | -0.22 – 0.25 | .884 |
| Trial × Run | 0.00 | -0.05 – 0.05 | .962 |
| **Random Effects** |  |  |  |
| σ^2^ | 0.39 |  |  |
| τ_00 Subject_ | 0.95 |  |  |
| ICC | 0.71 |  |  |
| N _Subject_ | 34 |  |  |
| Observations | 533 |  |  |
| Marginal R^2^ / Conditional R^2^ | 0.000 / 0.710 |  |  |

**Supplementary file 1E.** Mixed-effect model results for the effect of runs and trials on the pain ratings.

|  | **Pain ratings** | | |
| --- | --- | --- | --- |
| *Predictors* | *Estimates* | *CI* | *p* |
| (Intercept) | 78.58 | 75.30 – 81.85 | <.001 |
| Trial | 0.01 | -0.40 – 0.42 | .965 |
| Run | 1.98 | -0.91 – 4.87 | .179 |
| Trial × Run | -0.03 | -0.61 – 0.54 | .907 |
| **Random Effects** |  |  |  |
| σ^2^ | 59.34 |  |  |
| τ_00 Subject_ | 56.91 |  |  |
| ICC | 0.49 |  |  |
| N _Subject_ | 34 |  |  |
| Observations | 533 |  |  |
| Marginal R^2^ / Conditional R^2^ | 0.007 / 0.493 |  |  |

**Supplementary file 1F.** Mixed-effect model results for the effect of the FEPS expression scores and the pain ratings on the logarithmic transformed FACS scores.

|  | **Log(FACS scores + 1)** | | |
| --- | --- | --- | --- |
| *Predictors* | *Estimates* | *CI* | *p* |
| (Intercept) | 1.00 | 0.82 – 1.17 | <.001 |
| FEPS expression scores | 0.62 | 0.54 – 0.70 | <.001 |
| Pain ratings | 0.02 | -0.07 – 0.11 | .630 |
| FEPS expression scores × Pain ratings | -0.09 | -0.17 – -0.01 | .025 |
| **Random Effects** |  |  |  |
| σ^2^ | 0.29 |  |  |
| τ_00 Subject_ | 0.25 |  |  |
| τ_11 Subject.Rating_ | 0.03 |  |  |
| *ρ*_01 Subject_ | 0.56 |  |  |
| ICC | 0.49 |  |  |
| N _Subject_ | 34 |  |  |
| Observations | 533 |  |  |
| Marginal R^2^ / Conditional R^2^ | 0.396 / 0.690 |  |  |
